# World Endometriosis Research Foundation Endometriosis Phenome and Biobanking Harmonisation Project: IV. Tissue collection, processing, and storage in endometriosis research

**DOI:** 10.1016/j.fertnstert.2014.07.1209

**Published:** 2014-11

**Authors:** Amelie Fassbender, Nilufer Rahmioglu, Allison F. Vitonis, Paola Viganò, Linda C. Giudice, Thomas M. D’Hooghe, Lone Hummelshoj, G. David Adamson, Christian M. Becker, Stacey A. Missmer, Krina T. Zondervan, WERF EPHect Working Group, G.D. Adamson, C. Allaire, R. Anchan, C.M. Becker, M.A. Bedaiwy, G.M. Buck Louis, C. Calhaz-Jorge, K. Chwalisz, T.M. D'Hooghe, A. Fassbender, T. Faustmann, A.T. Fazleabas, I. Flores, A. Forman, I. Fraser, L.C. Giudice, M. Gotte, P. Gregersen, S.-W. Guo, T. Harada, D. Hartwell, A.W. Horne, M.L. Hull, L. Hummelshoj, M.G. Ibrahim, L. Kiesel, M.R. Laufer, K. Machens, S. Mechsner, S.A. Missmer, G.W. Montgomery, A. Nap, M. Nyegaard, K.G. Osteen, C.A. Petta, N. Rahmioglu, S.P. Renner, J. Riedlinger, S. Roehrich, P.A. Rogers, L. Rombauts, A. Salumets, E. Saridogan, T. Seckin, P. Stratton, K.L. Sharpe-Timms, S. Tworoger, P. Vigano, K. Vincent, A.F. Vitonis, U.-H. Wienhues-Thelen, P.P. Yeung, P. Yong, K.T. Zondervan

**Affiliations:** a Organ Systems, Department of Development and Regeneration, KU Leuven, Leuven, Belgium; b Department of Obstetrics and Gynecology, Leuven University Fertility Center, University Hospital Leuven, Leuven, Belgium; c Wellcome Trust Centre for Human Genetics, University of Oxford, Oxford, United Kingdom; d Department of Obstetrics, Gynecology, and Reproductive Biology, Brigham and Women's Hospital and Harvard Medical School, Boston, Massachusetts; e Boston Center for Endometriosis, Boston Children's Hospital and Brigham and Women's Hospital, Boston, Massachusetts; f Obstetrics and Gynecology Unit, San Raffaele Scientific Institute, Milano, Italy; g University of California San Francisco, San Francisco, California; h World Endometriosis Research Foundation (WERF), London, United Kingdom; i Palo Alto Medical Foundation Fertility Physicians of Northern California, Palo Alto, California; j Nuffield Department of Obstetrics and Gynaecology, University of Oxford, Oxford, United Kingdom; k Endometriosis Care Centre Oxford, University of Oxford, Oxford, United Kingdom; l Channing Division of Network Medicine, Department of Medicine, Brigham and Women's Hospital and Harvard Medical School, Boston, Massachusetts; m Department of Epidemiology, Harvard School of Public Health, Boston, Massachusetts

**Keywords:** Endometriosis, standardization, standard operating procedures, tissue, EPHect

## Abstract

**Objective:**

To harmonize standard operating procedures (SOPs) and standardize the recording of associated data for collection, processing, and storage of human tissues relevant to endometriosis.

**Design:**

An international collaboration involving 34 clinical/academic centers and three industry collaborators from 16 countries on five continents.

**Setting:**

In 2013, two workshops were conducted followed by global consultation, bringing together 54 leaders in endometriosis research and sample processing from around the world.

**Patient(s):**

None.

**Intervention(s):**

Consensus SOPs were based on: 1) systematic comparison of SOPs from 24 global centers collecting tissue samples from women with and without endometriosis on a medium or large scale (publication on >100 cases); 2) literature evidence where available, or consultation with laboratory experts otherwise; and 3) several global consultation rounds.

**Main Outcome Measure(s):**

Standard recommended and minimum required SOPs for tissue collection, processing, and storage in endometriosis research.

**Result(s):**

We developed “recommended standard” and “minimum required” SOPs for the collection, processing, and storage of ectopic and eutopic endometrium, peritoneum, and myometrium, and a biospecimen data collection form necessary for interpretation of sample-derived results.

**Conclusion(s):**

The EPHect SOPs allow endometriosis research centers to decrease variability in tissue-based results, facilitating between-center comparisons and collaborations. The procedures are also relevant to research into other gynecologic conditions involving endometrium, myometrium, and peritoneum. The consensus SOPs are based on the best available evidence; areas with limited evidence are identified as requiring further pilot studies. The SOPs will be reviewed based on investigator feedback and through systematic triannual follow-up. Updated versions will be made available at: http://endometriosisfoundation.org/ephect.

**Discuss:** You can discuss this article with its authors and with other ASRM members at **http://fertstertforum.com/fassbendera-werf-ephect-iv/**

The molecular analysis of tissue samples is an important approach to understand biologic changes that precede, or are a consequence of, disease. In cancer, molecular profiling of tumor versus normal tissues (e.g., the identification of oncogenic mutations in DNA through genomic sequencing, or of tumor-specific alterations in gene transcription patterns) has been fundamental to uncovering subtypes of disease 1, 2, 3 that may show differential response to treatment, thus paving the way for tumor-targeted personalized medicine to become a reality 4, 5, 6, 7. Indeed, initiatives such as the Cancer Genome Atlas (http://cancergenome.nih.gov) use large-scale profiling methodology to uncover cancer subtypes and further subtype specific treatment development (8). Other examples that recognize the value of large-scale molecular profiling initiatives to further translational medicine in general include the National Institutes of Health (NIH) Roadmap Epigenomic Mapping Consortium (9), focusing on developing epigenomic maps for a variety of normal human cell and tissue types (not endometrium), and the Genotype-Tissue Expression (GTEx) project that aims to establish a resource database and associated tissue bank for the scientific community to study the relationship between genetic variation and gene expression in a variety of human tissues (10). Thus, the collection/banking of tissues relevant to a disease from case and control subjects is a key investment that allows future research into pathogenesis, biomarkers, and the identification of novel drug targets.

Many centers worldwide have been collecting tissue samples—particularly ectopic and eutopic endometrium—from women with and without endometriosis, for a variety of research purposes 11, 12. One center, the University of California San Francisco NIH Human Endometrial Tissue and DNA Bank (http://obgyn.ucsf.edu/crs/tissue_bank/), has been collecting endometrium with the use of well described standard operating procedures (SOPs) specifically to allow collaborative research (12). However, variation in protocols between centers used for tissue collection, processing, and storage, is likely to result in bias and measurement error, and limits comparisons of results obtained in different studies and laboratories, as well as fruitful collaboration between centers. A crucial step that allows multicenter data to be combined and enables technical reproducibility of results between laboratories is the optimization of sample quality and minimization of variability due to nonbiologic (artefactual) processes. In addition, coordinated and detailed phenotypic characterization of research participants providing the specimens is a critical aspect without which collaborative analyses and validation/replication of results is not possible 13, 14. The adoption of validated internationally agreed-on SOPs for tissue sample collection, processing, and storage, and standardized phenotypic and other patient data collection, are crucial to optimize sample quality, reduce variability, and enable cross-center studies 15, 16, 17 and was identified as a key priority area in the 2008 and 2011 Endometriosis Research Directions Workshops 18, 19.

The mission of the World Endometriosis Research Foundation (WERF) Endometriosis Phenome and Biobanking Harmonisation Project (EPHect) is to develop a consensus on standardization and harmonization of phenotypic surgical/clinical data and biologic sample collection methods in endometriosis research. Specifically, to facilitate large-scale internationally collaborative, longitudinal, epidemiologically robust, translational, biomarker, and treatment target discovery research in endometriosis, EPHect provides evidence-based guidelines on: 1) detailed surgical and clinical and epidemiologic phenotyping (phenome) data to be collected from women with and without endometriosis to allow collaborative subphenotype discovery and validation analyses; and 2) SOPs for collection, processing and long-term storage of biologic samples from women with and without endometriosis. To the best of our knowledge, this harmonization initiative is unique in its scope—addressing standardization of phenotypic data collection and biologic sampling procedures simultaneously for a specific disease—with consensus reached from a large number of academic as well as industrial leaders in endometriosis research.

The development of the EPHect surgical (EPhect SSF and MSF) (13) and clinical questionnaires (EPHect EPQ-S and EPQ-M) (14) for robust phenotypic data collection and evidence-based SOPs for biologic fluid specimens (20) were described in the three previous papers in this series. In the present, final, paper we describe the development of evidence-based SOPs for the collection, processing, and storage of four tissues relevant to endometriosis research: ectopic and eutopic endometrium, peritoneum, and myometrium.

## Methods

**Figure 1:**
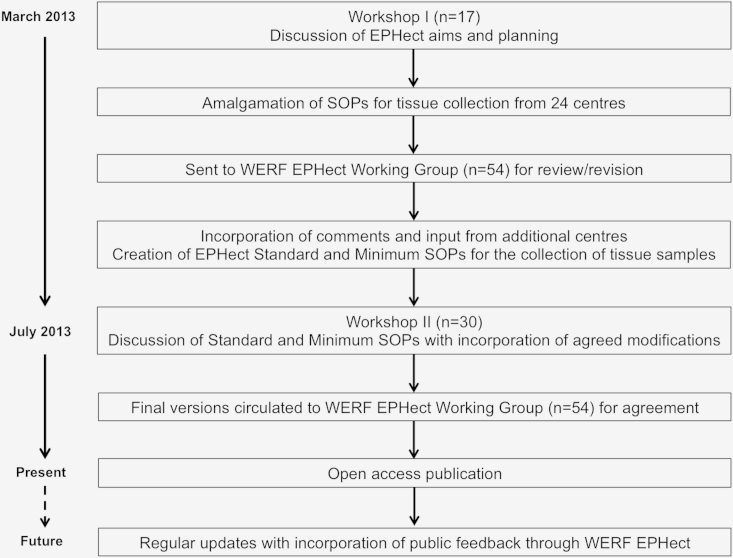
Flow diagram depicting the World Endometriosis Research Foundation Endometriosis Phenome and Biobanking Harmonisation Project (EPHect) development and consensus process (tissue sample standard operating procedures (SOPs)).

We conducted two workshops in March and July 2013, followed by several rounds of expert review, bringing together 54 leaders in endometriosis research and sample processing from 34 clinical/academic centers and three industry collaborators in 16 countries, to develop and reach consensus on evidence-based phenome collection and SOP guidelines (Fig. 1). During Workshop I and a subsequent consultation round, we identified 24 centers around the globe that collect tissues from endometriosis case and control subjects on a large scale (publication on >100 cases). We identified four tissue types (ectopic endometrium, eutopic endometrium, myometrium, and peritoneum) that were collected by the centers (Table 1); all provided SOPs for sample collection, processing, and storage.

**Table 1:** Number of centers contributing to the Endometriosis Phenome and Biobanking Harmonisation Project that have collected tissue for the purpose of endometriosis research (ectopic endometrium, eutopic endometrium, myometrium, and/or peritoneum).

Sample type	No. of centers
Ectopic endometrium	16
Eutopic endometrium	22
Myometrium	5
Macroscopically normal-appearing peritoneum	8

In addition to the information provided by the 24 centers, we searched for publicly available SOPs from general large-scale biobanking efforts (UK Biobank) and large biorepositories (International Society for Biological and Environmental Biorepositories, National Cancer Institute-Biorepositories and Biospecimen Research Branch, Australian Biospecimen Network), and conducted a systematic literature search in Pubmed for English-language publications describing crucial steps in SOPs, with the use of the search terms: “standard operating procedure” AND (“endometriosis” or “tissues” or “endometrium” or “myometrium” or “peritoneum” or “best practice” or “biobank”). Reference lists of retrieved papers were hand searched for additional references and material. Furthermore, other online material from biobanks and biorepositories was sought with the use of the Google search engine with the same search terms. On the basis of this information, we compiled draft consensus SOPs, identifying steps that varied between center-specific SOPs, but for which little 12, 15 or no evidence could be obtained. Before Workshop II, consensus documents and associated evidence and queries were distributed to the WERF EPHect Working Group, which again included external consultants outside the endometriosis research field with relevant tissue banking expertise. During Workshop II, and a separate e-mail consultation process among those who were unable to attend the workshop, the final consensus SOPs were reviewed and agreed on (Supplemental Table 1 and Supplemental Appendices 1–4, available online at www.fertstert.org).

Although validity, reliability, and scientific advancement are the main goals of WERF EPHect, an important point acknowledged by the EPHect Working Group was that there are likely to be differences in resources and logistics among centers that may mean they are unable to adhere to some of the strictest standards in the procedures. All experts therefore agreed on two tiers for most steps in the SOPs: “standard recommended” and “minimum required.” We strongly advise “standard recommended” SOPs to be adopted where possible, because they will yield results that are least prone to variation and degradation of the samples; the “minimum required” SOP steps are offered to provide the fundamentals for standardization that need to be adhered to as an absolute minimum requirement, given unavoidable logistical and budgetary circumstances. It is important to note that publication of results generated with the use of samples collected following the WERF EPHect SOPs need to state which EPHect procedures were used and explicitly state in publications any alterations made. Per scientific method, we strongly recommend that each center maintain a copy of the details of the exact protocol used.

When collecting biologic samples for research purposes, additional data items need to be collected to allow interpretation of results from the samples (20). For this purpose, the EPHect Working Group developed a consensus EPHect Biospecimens Form (Supplemental Appendix 5, available online at www.fertstert.org) to be completed at each sample collection event.

Approval by an Ethics Committee or Institute Review Board was not required for formation of the EPHect Working Group, review of existing literature, or consensus regarding best practices for endometriosis research described within the WERF EPHect four-manuscript series. This endeavor did not include data from human subjects.

## Results

Below we describe the rationale behind the development of the WERF EPHect SOPs for collection, processing, and storage and the evidence used in their construction. Although methods of collection and labeling of different tissues are distinct, a considerable number of steps in the different SOPs are identical across tissue types, relating to: 1) time and temperature between collection, transport, and storage; 2) processing procedure; 3) type of storage container; 4) storage conditions; and 5) aspects of long-term storage.

### Methods of Collection

#### Ectopic endometrium

Ectopic endometrium (endometriotic foci/disease) is excised with the aid of cold scissors/scalpels, electrosurgery, harmonic scalpel, or laser (CO_2_, Nd:YAG, and others) (13). The presence of stromal and glandular epithelial cells (as well as inflammatory cells and percentage fibrotic or fibromuscular component) should be verified histologically by an experienced pathologist. Pathologic analysis of the tissues accrued before either freezing or release for research is necessary to document the histologic characteristics of the tissues (percentage glands, stroma, and inflammation); histology slides must be prepared in a cryostat at low temperatures to maintain the integrity of the molecules in the tissue. Ectopic endometrium can be snap frozen in liquid nitrogen (LN_2_), placed in an RNA-stabilizing/preservation solution, or fixed. The collection least likely to cause alterations to the molecular composition of the tissue, and most versatile in terms of downstream analyses possibilities, is sharp dissection without heat to avoid “heat artifacts” (13), followed by snap freezing in LN_2_ and long-term storage in a −80°C freezer or ideally in a LN_2_ freezer. If not immediately snap frozen, samples must be kept on ice (4°C) from the time of collection and transported to the research laboratory for immediate (within 15 minutes) processing and storage. Samples intended for tissue culture should be transferred to cold (4°C) tissue culture medium (not buffer) while awaiting processing.

#### Eutopic endometrium

Five methods can be used to collect eutopic endometrium: 1) an endometrial sampling device; 2) curettage with cervical dilation, if necessary; 3) hysteroscopic resection; 4) post-hysterectomy excision; and 5) brushing (13). An endometrial sampling device is a thin plastic tube that is inserted into the uterus and used to collect endometrial samples. Samples are collected by aspirating the tissue into the plastic tube and then transferring the aspirated tissue to a storage container (12). During a curettage, strips of endometrium are scraped off the uterine lining with the use of a curette. However, this approach may not be suitable for morphologic or histologic evaluation, because the procedure can damage the tissue (K. Timms, personal communication). In the case of hysterectomy specimens, eutopic endometrium can be taken with the aid of a knife or scissors; whereas a hysteroscopy specimen is taken with the use of curette, scissors, endometrium sampling device, or resectoscope using monopolar or bipolar energy. To avoid heat artifacts, tissue collection with the use of electrosurgery is not recommended. For the brushing method, a Tao brush (Cook) is used to obtain samples by insertion into the uterine cavity with a plastic sheath covering the brush. Once inserted, the sheath is withdrawn and the brush is rotated through 360 degrees clockwise and then counterclockwise (21). Brushing is more expensive but likely to be less painful for patients (21). Whichever collection method is used, ideally the menstrual phase should be determined from an endometrium sample by an experienced pathologist, in addition to recording the first day of the last menstrual period, because this information is required for subsequent data interpretation.

#### Myometrium

Myometrial tissue of women with and without endometriosis is used in in vitro studies, such as gene expression assays and histologic examination of, e.g., nerve fibers (22). The myometrium is excised with the aid of diathermy/laser (CO_2_, Nd:YAG, and others) or cold scissors/scalpels (13) and can be snap frozen, placed in an RNA-stabilizing/preservation solution, or fixed. The preferred method is sharp dissection without the use of heat, to avoid “heat artifacts” (13). For hysterectomy specimens, the myometrium is obtained with the aid of scalpel or scissors. The myometrial tissue can also be excised by TruCut biopsy (Carefusion), which may be more representative of the deeper layers (23).

#### Peritoneum

Peritoneal tissue has been used to investigate the pathogenesis of endometriosis mainly to understand the passive or active contribution of the peritoneum to the establishment and growth of ectopic endometrium 24, 25. A peritoneal biopsy can be taken from an “endometriosis-prone” site (e.g., within the pouch of Douglas) and another from a site less prone to harbor endometriosis, such as the lateral/pelvic brim or the anterior abdominal wall (13). Collection sites should be recorded with the use of the WERF EPHect SSF (13). Samples can be obtained with the use of either brushing or surgical devices. Surgically, peritoneum is excised with the aid of electrosurgery, ultrasound energy, harmonic scalpel, laser (CO_2_, Nd:YAG, and others) or cold scissors/scalpel. The best method is sharp dissection without heat, to avoid “heat artifacts.” Alternatively, with the use of a Tao brush (Cook), a sweep is made over the peritoneal surface, as described in more detail in the supplemental SOPs (Supplemental Appendix 4). The Tao brush is useful for the collection of peritoneal mesothelial cells for cell culture. The size of the peritoneal biopsies should be determined by the research question within parameters that are clinically justifiable. In general, the sample contracts significantly after excision.

### Sample Quality: Time and Temperature between Collection and Storage

The time between surgical tissue extraction and storage needs to be as short as possible (12). In the EPHect SOPs, we recommend limiting this to 15 minutes to minimize enzymatic degradation. Although genomic DNA is relatively stable (26), mRNA is particularly sensitive to degradation by abundant and ubiquitous RNAses (microRNA may be more stable than mRNA) 27, 28; phosphoproteins are also unstable, underscoring the need for rapid processing (29).

The effect of tissue ischemia on RNA analysis with the use of gene expression microarray analysis has been documented for a variety of human tissues 30, 31, 32, 33, 34, 35, 36, and there is broad consensus on the importance of standardized tissue procurement procedures and minimizing processing time until freezing (12). Sheldon et al. demonstrated high-quality RNA for microarray analysis if the time between collection and preservation did not exceed 10 minutes (12). Others showed that 15 minutes after surgery 10%–15% of all detectable genes and proteins, and after 30 minutes 20%, differed significantly from the baseline values (30). Therefore, the time between tissue extraction and freezing (cold ischemia time) must be recorded and made available to investigators requesting tissues for research. Depending on the molecule(s) of interest, longer cold ischemia times may be appropriate. In general, it is considered to be best practice to minimize cold ischemia time as much as possible.

Investigators need to consider carefully what the samples are to be used for and adapt the logistical set-up of their study to meet the SOP requirements. If there will be a longer delay in processing than recommended, then pilot studies should be conducted to test the stability of individual biomarkers, because some biomarkers are stable for up to 48 hours 37, 38. Particular attention should be paid to the temperature conditions during the time between excision of the tissue and its preservation (12), because these can have a major impact on the quality of samples (39). The WERF EPHect SOPs recommend using precooled transport/fixation media (where applicable) and keeping samples on ice (4°C) at all times before storage to allow utility of the samples in assays that are both sensitive and insensitive to room temperature conditions. For studies of highly unstable molecules, such as RNA, immediate snap freezing in LN_2_, as described in the next section, is recommended, or alternatively stabilization of RNA in appropriate stabilizing/preservation solution, which allows the sample to be temporarily kept at temperatures as high as 37°C before long-term freezing. The time between tissue extraction and storage should be recorded.

### Processing and Storage

The choice of processing via: 1) immediate snap freezing in LN_2_, 2) immersion in an RNAse inhibitor solution followed by freezing or paraffin embedding, 3) Neutral buffered formalin fixation/universal molecular fixative and paraffin embedding (FFPE); or 4) in vitro culture depends on a number of factors, including the anticipated future use of samples, amount of tissue available, and budgetary constraints. Table 2 presents the downstream uses allowed by the different processing and storage methods. Note that when there is an interest in conducting analyses on a cellular subtype of ectopic or eutopic endometrium (which can be highly heterogeneous and can include epithelial cells, stromal cells, fibrotic tissue, muscle tissue, and blood in addition to the endometrial glands of interest), laser capture microdissection can be performed on tissues stored in a variety of ways, including in an RNA stabilizing/preservation solution 40, 41, fresh frozen, or FFPE (42). The choice of storage containers depends on the molecular outcome of interest (e.g., some plastics retain proteins that could interfere with proteomic analyses; nonsterile tubes would not be suitable for most studies, particularly those of RNA).

**Table 2:** Downstream uses of tissue according to different processing and storage methods.

	Cell isolation/culture	DNA	Metabolites	Protein	RNA
Snap frozen tissue		✓	✓	✓	✓
Frozen viable cells	✓	✓	✓	✓	✓
RNAse inhibitor^a^		✓		✓	✓
Fixed tissue		✓	✓	✓	✓
Fresh tissue	✓	✓	✓	✓	✓

a Commercially available products: Allprotect Tissue Reagent (Qiagen); DNA/RNA Shield (Zymoresearch); ProtectRNA (Sigma-Aldrich); Ribolock (Thermoscientific); RNAlater (Qiagen); Ambion RNAsecure Reagent (Life-technologies); SUPERase-In (Life-technologies); PAXgene Tissue Containers (Qiagen).

DNA is very stable, and recoverable from samples treated and stored with the use of a range of methods (fresh frozen or fixed), although DNA recovered from long-term archived FFPE samples is compromised regarding strand length 43, 44 owing to the cross-linking properties of formalin. Severely fragmented DNA may have consequences for technologic applications, such as long-read next-generation DNA sequencing; but even in situations where stored DNA is fragmented, methodologies to accurately sequence such samples are improving continuously, as demonstrated by the extreme example of the successful sequencing of ancient DNA (45). Multiple studies have investigated optimal processing and storage conditions for tissues for RNA extraction, with RNA quality most commonly determined by RNA integrity number 46, 47, 48, 49, 50, 51, 52. Because RNA degrades rapidly after tissue collection, best storage methods are immediate snap freezing in LN_2_ or immediate immersion in stabilizing/preservation solution 46, 47, 48, for which a range of commercially available options exist (Table 2). Another option is to use a universal molecular fixative, which yielded high-quality RNA from paraffin-embedded tissue in one study (49). Two studies showed no loss of RNA quality in normal RNAlater-treated tumor tissues for up to 7 days at room temperature 46, 47. Tissue thickness is crucial for successful RNA stabilization to enable rapid diffusion of the RNA stabilizing/preservation solution or fixative. To ensure rapid and reliable stabilization of RNA in the entire tissue, it is recommended that the sample is cut into slices not thicker than 0.5 cm. A study comparing immediate snap freezing in LN_2_ with ethanol-fixation and RNAlater-preservation techniques for subsequent assessment of gene expression changes in cervical cancer showed RNA quality to be equivalent to or better in snap-frozen tissues than RNAlater- or formalin-fixed preserved tissues (50). A recent study has investigated the preservation of nucleic acids in paraffin-embedded clinical tissue samples fixed to Z7, RCL2, PAXgene, Allprotect, and RNAlater compared with preservation with the use of LN_2_ or formalin (51), suggesting that the PAXgene tissue system provided the best alternative, enabling high-quality molecular analyses, immunohistochemistry, and sufficient morphologic examination.

In situations where long-term storage of tissue samples is required, and there is limited immediate access to LN_2_ or −80°C freezers, paraffin embedding of samples after preservation/fixing is an obvious choice. FFPE tissue is not considered to be ideal for RNA and some DNA applications (53). Many studies have investigated the impact of prolonged formalin fixation 54, 55, 56, 57, 58, 59, 60, 61 and recommended that formalin fixation of specimens should be rapid and not delayed for more than 1 hour 54, 60, 61. Alternatively, glutaraldehyde is another cross-linking agent for tissue fixation that better preserves high-molecular weight DNA compared with 10% formalin (62). Although widely used as a fixative for standard electron microscopy, the slow penetration and the need for periodic purification have greatly limited its use as a biologic fixative (62).

The most common method of preservation and storage of tissues to be used for proteomic and metabolomic research has been snap freezing in LN_2_, although some studies have been conducted to investigate the effect of stabilizing/fixing methods on proteomic data quality. Recent studies have shown that FFPE tissue samples are suitable for proteomic analysis (63), and that there is no significant difference in the number of proteins identified from fixed versus frozen tissues, even with prolonged storage (63). One study assessed the patterns of protein abundance in one-dimensional gels for five tissues of the gulf killifish after snap-freezing tissues in LN_2_ or immersion of fresh tissues in RNAlater, showing no differences between the two methods (64). Another study compared the quality of DNA, protein, and RNA extraction from RNAlater and snap freezing (65). Those authors concluded that RNAlater is an excellent storage agent for yeast cells and, most likely, for other cell types and tissues, to preserve the samples for major “omics” techniques and microscopic analyses (65).

Given the evidence provided, the EPHect SOPs advise, where LN_2_ or −80°C freezers are available, to snap freeze tissues as soon as possible after collection (within 15 minutes for RNA analysis) or otherwise immerse in RNA-stabilizing solution, followed by freezing if molecular analysis is to be conducted in the future. Only if freezing for long-term storage is not an option, or where large volumes of tissue allow multiple storage methods, archiving of FFPE samples following the relevant EPHect SOP is recommended.

### Long-term Storage

Stability studies for a range of biomolecules have shown that samples should be stored, as a minimum requirement, in −80°C freezers, if there is no access to LN_2_ freezers (26). Studies have shown that depending on the location of the sample in mechanical −80°C freezers, temperature can fluctuate from −90°C to −43.5°C (66). LN_2_ freezers are much colder and have less temperature variability than −80°C mechanical freezers (67). However, LN_2_ freezers are much more expensive to maintain than −80°C mechanical freezers and require access to a regular LN_2_ source. If many LN_2_ freezers are placed in the same room, then it is crucial to have oxygen sensors in the room that will alarm if oxygen levels dip into an unsafe range. An alternative to LN_2_ freezers is −150°C cryogenic freezers.

It is crucial to manually check the −80°C freezers at least twice a week for temperature variations, and to also monitor ambient temperature of the biobanking facility. In addition, every freezer should be equipped with an alarm system to give notification (via phone, text, or e-mail) in case of temperature fluctuations according to local practice. As additional safety measures it is important to: 1) have an empty back-up freezer available if possible; and 2) in situations where tissue samples are sufficiently large to be cut and stored in multiple pieces, ensure these are stored in separate freezers. Recommended also is the purchase of dual compressor freezers which allow them to continue working and maintaining low temperatures in case one of the compressors fails, and freezers that are connected to battery back-ups (to protect them from power fluctuations). Importantly, it is recommended that these freezers are connected to a power generator that can ensure continued power in case of an emergency, and that a detailed emergency plan be developed with clear responsibilities and duties in case samples need to be moved to the back-up freezer because of power failures or freezer malfunctions.

The duration of long-term storage before analysis also is a potentially important consideration for the stability of biomolecules; however, there is limited evidence on the extent of these effects. The effect of long-term storage on RNA stability has been studied mainly in recent small-scale pilot studies of tumor tissues: 1) Olivieri et al. showed that storage of head/neck tumors at −140°C up to 7 years did not significantly change the quality of RNA measured by RNA integrity number scores (68); 2) Bao et al. showed that colon cancer tissue snap frozen in liquid nitrogen and then stored at −80°C up to 3 years did not significantly influence RNA quality (69); and 3) Shabihkhani et al. reviewed the limited evidence from currently published pilot studies, showing that tissue storage at −80°C can preserve DNA and protein for years but RNA can show degradation within 5 years (70). Regarding endometriosis-related tissues, there is a need for pilot studies that investigate the effect of long-term storage in −80°C versus LN_2_ freezers for different time intervals to provide guidelines for long-term storage limits of tissues for RNA analysis.

### Biospecimens Form

The EPHect Biospecimens Form (Supplemental Appendix 5) has been discussed in detail in Rahmioglu et al. (20). It includes items that the WERF EPHect Working Group agreed were essential data that need to be recorded and collected from the participant when collecting biologic samples. Relevant to tissue samples are: menstrual data, medication use, and excision method(s) for biopsies. The first day of the last menstrual period as well as general menstrual characteristics (e.g., cycle length, regular versus irregular cycle) are crucial to record, because various molecules other than DNA are likely to be expressed at different levels in different phases of the menstrual cycle, as demonstrated in gene expression studies 71, 72, 73, 74, 75. Ideally, the first day of the next menstrual period should also be recorded, because forward-dating for the luteal phase is more accurate than back-dating 76, 77. Medication details also are important to document when collecting tissues, to allow testing of the sensitivity of study results to medication use. Note that hormone use, which is critical information when interpreting results from tissues such as endometrium, is collected as part of the WERF EPHect patient questionnaire (14). The WERF EPHect Working Group agreed that recording of height, weight, and waist and hip circumferences is important, owing to the consistent phenotypic and genetic associations of obesity and fat distribution traits in endometriosis 14, 78 and recommends following National Health and Nutrition Examination Survey III guidelines (adapted from World Health Organization guidelines) for standardization of their measurement 79, 80; more details are given by Rahmioglu et al. (20).

## Discussion

We have presented WERF EPHect consensus SOPs for the collection, processing, and storage of tissues, together with a short Biospecimens Form for collecting additional data necessary for informative analysis of the samples. This consensus was developed and agreed on by 34 clinical/academic institutions and three industry collaborators from 16 countries across five continents. We believe it represents a groundbreaking opportunity for endometriosis research centers that will allow for more meaningful cross-center collaborations, consistency in data interpretation, and an increased probability for discovery of reliable diagnostic biomarkers.

The SOPs presented focus on the tissue collection processes required for high-quality downstream analysis of biomolecules. Other specific downstream applications, such as the quantification of specific environmental chemicals in the samples, are likely to require different collection materials as well as the adaptation of SOPs. In addition, we recommend a number of areas where pilot experiments may be needed to investigate the effect of the parameters involved in these steps. For example, there is no firm evidence-based consensus on the ideal time period between tissue excision until storage, which is likely to vary depending on the molecule of interest. Our standard recommended guideline is based on evidence from studies of mRNA, a molecule known to display very rapid degradation; other molecules may be much more stable over time. Furthermore, it would be useful to conduct pilot studies on the optimal amount of tissue to be collected for different purposes, the influence of different methods of surgical collection, the influence of different temperature conditions between excision and storage, the influence of phosphate-buffered saline solution rinsing on the endometrium before freezing, and the tissue-specific levels of expression of the molecule(s) of interest to the investigator.

All questionnaires and SOPs produced by the WERF EPHect Working Group are freely available for use by investigators, subject to signed written informed consent obtained from each patient and local ethical approval for the study according to ethical principles for clinical research as summarized in the Declaration of Helsinki. To enable the multicenter collaborations envisaged by the WERF EPHect initiative, it is essential that centers adopting the WERF EPHect instruments and SOPs ensure that patients provide informed consent that allows their data and biologic samples to be used in future multicenter (inter)national collaborations, and that appropriate Ethics Committee and Institute Review Board approval is obtained that allows for such collaborations. The evidence base for all EPHect data collection instruments and SOPs will be reviewed continuously with feedback provided by investigators and through systematic surveys and follow-up reviews after 1 year and then every 3 years. Therefore, investigators are strongly encouraged to provide feedback, suggestions for the SOPs, and additional procedures not covered in this article. Updates of instruments will remain freely accessible to the research community through the WERF EPHect website (http://endometriosisfoundation.org/ephect). Investigators are asked to cite WERF EPHect SOPs in their publication if they use the SOPs (either “minimum required” or “recommended standard”) and, if they diverge, to note the specific modifications to their procedures. We recommend that each center maintain a copy of the exact protocol used.

The availability of stored tissue samples collected, processed, and stored according to standardized procedures—and for which detailed, globally harmonized, surgical and clinical phenotypic data also have been recorded—will allow high-quality collaborative research involving large sample sizes that is enriched by the inclusion of multiple sites possessing phenotypic subpopulations at a scale that is unprecedented in the field of endometriosis. We believe that the ability to attribute observed differences to true disease heterogeneity and not protocol variation is exceptionally valuable and of paramount importance for valid scientific discovery in endometriosis. In the next phase of the EPHect initiative, WERF aims to: 1) develop freely available stand-alone applications as well as web-based systems to facilitate center-restricted data entry and reduce costs and time expenditure to individual centers; and 2) amalgamate a voluntary registry of centers using EPHect data collection tools and biologic sample SOPs that would offer any investigator a transparent platform for the establishment of new collaborations. We hope that the EPHect initiative will inspire existing as well as new investigators in the field of endometriosis to join forces and work together to improve our understanding of this poorly understood heterogeneous disease and provide new effective methods for its diagnosis and treatment.

## Supplemental data

Supplemental Table 1

Supplemental Appendices 1–5
