# Supplementary material for: World Endometriosis Research Foundation Endometriosis Phenome and Biobanking Harmonisation Project: IV. Tissue collection, processing, and storage in endometriosis research
Source: Fertil Steril. 2014 Nov;102(5):1244–53. doi: 10.1016/j.fertnstert.2014.07.1209 (PMC4230778; doi:10.1016/j.fertnstert.2014.07.1209)
Supplement: Supplemental Table 1 [file mmc1.docx]

| **PROTOCOL STEPS** | **All sample types** | | | | | | | |
| --- | --- | --- | --- | --- | --- | --- | --- | --- |
| **Sample collection meta-data (incl. excision method)** | See Becker et al. (ref), and complete biospecimen questionnaire (Appendix V) | | | | | | | |
| **Preparation** | Prepare and label vials/tubes for transfer and storage of samples as much as possible | | | | | | | |
| **Specimen transfer to the laboratory** | As soon as possible; keep on ice until preservation/storage;  If transport likely to take >15 mins, consider immediate snap-freezing or RNA stabilising procedures in/near theatre. | | | | | | | |
| ***SD****=Standard*  ***RM****=Required minimum* | **SD** | | | | **RM** | | | |
| **Sample**  **processing** | - Record time of starting sample processing - Rinse tissue with PBS (except for RNA studies) - Weigh tissue samples; if tissues to be stored in separate pieces, prioritize: 1^st^ snap freezing, 2^nd^ RNA stabilizing solution [see detailed protocol for examples of commercially available products], 3^rd^ universal molecular fixative or formalin fixation. | | | | - Record time of starting sample processing - Rinse tissue with PBS (except for RNA studies) - Weigh tissue samples; if tissues to be stored in separate pieces, prioritize: 1^st^ snap freezing, 2^nd^ RNA stabilizing solution [see detailed protocol for examples of commercially available products], 3^rd^ universal molecular fixative or formalin fixation. | | | |
|  | **Type of processing** | | | | | | | |
|  | **Fresh tissue** | | **Snap-frozen tissue** | | **RNA stabilising + freezing** | | **Fixed tissue** | |
| ***SD****=Standard*  ***RM****=Required minimum* | **SD** | **RM** | **SD** | **RM** | **SD** | **RM** | **SD** | **RM** |
|  | - Store in fluid media/PBS at 4°C (max 24 hrs); - Transfer to waterbath (37°C) for cell culture | - Store in fluid media/PBS at 4°C (max 24 hrs) | - Store in vial with screw top gasket; - Snap freeze in liquid nitrogen (LN_2_) immediately; - Record time. | - Store in vial with screw top gasket; - Snap freeze in LN_2_ as soon as possible; - Record time. | - - Within 15 mins of collection, cut samples to ≤0.5cm + immerse in RNA stabilising solution^1^ in screw top gasket vial. - Record time; - Store at 4°C for 24 hours prior to freezing. | - As soon as possible, cut samples to ≤0.5cm + immerse in RNA stabilising solution^1^ in screw top gasket vial. - Record time; - Store at 4°C for 24 hours prior to freezing. | - Within 15 mins of collection, mount flat in Histokinette^TM^ cassette in 20ml 10% NBF^2^; - Record time; Transport at room temperature or place on ice. - Leave in NBF for max 24 hours; - Transfer to graded alcohol solutions + paraffin embedding. | - Within 1 hr of collection, mount flat in Histokinette^TM^ cassette in 20ml 10% NBF^2^; - Record time. Transport at room temperature or place on ice; - Leave in NBF for max 24 hours; - Transfer to graded alcohol solutions + paraffin embedding. |

| ***SD****=Standard*  ***RM****=Required minimum* | **SD** | **RM** |
| --- | --- | --- |
| **Storage** | - Store in LN_2_ freezer. | - Store in -80°C or lower freezers. |
| **Freezer check** | - Alarm system setup on all freezers. - Biweekly human check. | - Biweekly human check. |
| **Data recording Checklist** | - Record protocol, specifying which steps are adhered to (standard or minimum). - For each sample, record:   - Date and time of collection (Date: __/__/__ and __:__am/pm).   - Start time of sample processing in the laboratory (Date: __/__/__ and __:__am/pm).   - Type and number of lesions prepared.   - Date and time sample is stored into freezer (Date: __/__/__ and __:__am/pm).   - Any variations or deviations from the SOP, problems, or issues.   - Any freeze-thaw event that occurs with a sample for any reason. - Keep a log of bi-weekly freezer checks. | |

^1^ Follow and record manufacturer’s protocol.

^2^ NBF= neutral buffered formalin.
