# Supplementary material for: World Endometriosis Research Foundation Endometriosis Phenome and Biobanking Harmonisation Project: IV. Tissue collection, processing, and storage in endometriosis research
Source: Fertil Steril. 2014 Nov;102(5):1244–53. doi: 10.1016/j.fertnstert.2014.07.1209 (PMC4230778; doi:10.1016/j.fertnstert.2014.07.1209)
Supplement: Supplemental Appendices 1–5 [file mmc2.docx]

**Supplemental Appendix I:

Detailed standard operating procedure for the collection, processing and storage of ectopic endometrium samples**

**NOTES**

- This SOP does not cover safety procedures for the collection and processing of these samples and personnel must follow institutional biosafety guidelines**.**
- For a summary version of this protocol with side-by-side standard vs. minimal protocol step comparisons, please see Supplemental Table 1.
- As this protocol applies to different processing and storage methods (e.g. the use of RNA stabilising solution; immediate snap freezing in theatre), keep a copy of the exact step-by-step protocol used in your lab.

***Processing and storage materials***

1. Biospecimen questionnaire (Supplemental Appendix V);
2. Log sheet to record sample-related data;

*Frozen tissues:*

1. Labels suitable for long-term freezer storage, and IDs printed using 2D barcoding.
2. Aliquot vials with screw top gasket closure; liquid nitrogen.
3. Buffers: phosphate buffered saline (PBS);
4. RNA stabilizer solution *(optional if planning RNA studies)*
5. Freezers: -80C or liquid nitrogen (LN_2_)

*Fixed tissues:*

1. Labels with IDs printed using 2D barcoding;
2. Neutral buffered formalin, universal molecular fixative, paraffin;
3. HistokinetteTM cassette

**1. Ectopic endometrium collection**

1.1 Pr_i_or to tissue collection at surgery, prepare as many of the materials required for storage of the samples and recording of the data (log sheet, storage vials and labels).

1.2. Preparation of vials/tubes for surgical collection *(NB. If samples are to be snap-frozen immediately in liquid nitrogen in/near theatre in their final storage vials, which is recommended, adopt the final labelling guidelines in section 2 of this protocol).*

1.2.1. ***Standard collection:*** Pre-label each collection vial with a 2D barcode in addition to a human readable unique identifier, participant ID, date of collection and type of sample. Record on the log sheet the date and time of sample collection (Date: __/__/__ and __:__am/pm).

1.2.2. ***Required minimum:*** Pre-label each collection vial with a unique identifier, participant ID, date of collection, and type of sample. Record on the log sheet the date and time of sample collection (Date: __/__/__ and __:__am/pm).

1.3. For guidance on surgical excision of ectopic endometrium biopsies, please refer to Becker *et al.,* (Becker *et al.*, 2014) and complete biospecimen questionnaire for each study recruit (Supplemental Appendix V).

1.4. Deposition of collected tissue samples.

1.4.1. ***Standard collection:*** Insert tissues in prepared, labelled vials/tubes and snap-freeze immediately in liquid nitrogen in/near theatre in their final storage vials.

1.4.2. ***Required minimum:*** Insert tissues in prepared, labelled vials/tubes and snap-freeze as soon as possible in liquid nitrogen in/near theatre in their final storage vials. If delays >15 minutes are expected, immerse in an RNA stabiliser solution if required.

**2. Sample processing**

2.1. Preparation of vials/tubes for collection and storage. Use labels and ink suitable for long-term freezer storage. Do not use laser printers or most ink-jet printers as the ink can crack and fall off the label when frozen at ultra-low temperature.

2.1.1. ***Standard collection****:* Pre-label the aliquot vials with the participant ID number followed by a unique aliquot ID number. Include date of sample creation and sample type. For example: ENDO-123456-U654321-EL: Center identifier (ENDO), participant ID (123456), unique aliquot vial ID (U654321), and sample type (EL for ectopic endometrium). Further, include the above information in human readable format and in a 2D barcode on the label.

2.1.2. ***Required minimum:*** Pre-label the aliquot vials with the participant ID followed by the sample aliquot number. Also include date of sample creation and sample type on the label. For example: ENDO-123456-EL-01: Center identifier (ENDO), participant ID (123456), type of sample (EL for ectopic endometrium), aliquot number (01).

2.2. Record time of starting sample processing on log sheet.

2.3. Rinse tissue with PBS (except for RNA studies).

2.4. Weigh tissue samples. If tissue size and weight allows storage in separate pieces, prioritize in the following order before freezing for long-term storage.: 1^st^ snap freezing, 2^nd^ RNA stabilizing solution followed by freezing, 3^rd^ universal molecular fixative or formalin fixation. *NB. If RNA is of key interest, and time to freezing is likely to take more than 5 minutes, consider immediate immersion in an RNA stabilizer solution as the first priority.*

2.5. Fresh Tissues

2.5.1. ***Standard collection****:* Fresh tissue can be stored in fluid (eg. media or PBS) up to 24 hours at 4°C. Transfer to waterbath (37°C) to keep cells alive for cell culture.

2.5.2. ***Required minimum*:** Fresh tissue can be stored in fluid (eg. media or PBS) up to 24 hours at 4°C.

2.6. Frozen Tissues

2.6.1. ***Standard collection****:* Tissue samples should be stored in an aliquot vial with screw top gasket closure. Snap freeze in liquid nitrogen within 15 minutes, record time, and transfer to freezers for long-term storage (see section 3).

2.6.2. ***Required minimum****:* Tissue samples should be stored in an aliquot vial with screw top gasket closure. Snap freeze in liquid nitrogen as soon as possible, record time, and transfer to freezers for long-term storage (see section 3).

2.7. RNA stabilizing solution

[commercially available products: Allprotect Tissue Reagent® (Qiagen, Venlo, Netherlands); DNA / RNA Shield™ (Zymoresearch, Irvine, USA); ProtectRNA™ (Sigma-Aldrich, St.Louis, USA); RiboLock™ (Thermoscientific, Waltham, USA); RNAlater® (Qiagen, Venlo, Netherlands); Ambion® [RNAsecure™ Reagent](http://products.invitrogen.com/ivgn/product/AM7006) [(Life-technologies, Carlsbad, USA); SUPERase•In™ (Life-technologies, Carlsbad, USA); PAXgene Tissue Containers (Qiagen, Venlo, Netherlands)]](http://products.invitrogen.com/ivgn/product/AM2694)

Before immersion in RNA stabilizing solution, cut large tissue samples to size according to manufacturer’s protocol.

2.7.1. ***Standard collection****:* Tissue samples should be stored in an aliquot vial with screw top gasket closure containing RNA stabilizing solution within 15 minutes of collection, and stored at 4°C for 24 hours prior to freezing (see section 3).

2.7.2. ***Required minimum****:* Tissue samples should be stored in an aliquot vial with screw top gasket closure containing RNA stabilizing solution, and stored at room temperature for 24 hours prior to freezing (see section 3).

2.8. Fixed Tissues

2.8.1. ***Standard collection****:* Tissue samples should be mounted flat in a Histokinette^TM^ cassette into 20ml of 10% neutral buffered formalin (NBF), within 15 minutes (record time). The sample is transported at room temperature or can be placed on ice. The tissue should remain in NBF for no more than 24 hours before transfer to graded alcohol solutions and paraffin embedding. Alternative fixatives include universal molecular fixative, which may better preserve RNA.

2.8.2. ***Required minimum****:* Tissue samples should be mounted flat in a Histokinette^TM^ cassette containing 20 ml of 10% neutral buffered formalin (NBF), within 1 hour (record time). The sample is transported at room temperature or can be placed on ice. The tissue should remain in NBF for no more than 24 hours before transfer to graded alcohol solutions and paraffin embedding. Alternative fixatives include universal molecular fixative, which may better preserve RNA.

**3. Storage and data recording**

3.1. Date and time of storage should be recorded on the log sheet. Also record the type, number of aliquots prepared.

3.1.1. ***Standard collection****:* Store samples in liquid nitrogen (LN_2_) freezers, as they have less temperature fluctuations than -80°C freezers.

3.1.2. ***Required minimum:*** Store samples in -80°C or lower freezers.

3.5. Record on the log sheet any variations or deviations from the SOP, problems, or issues (e.g. vial cracked during processing).

3.6. Record the location of each sample into the freezer including freezer number, rack, box, and spot in the box along with all other sample attributes in a database. If possible, avoid using a spreadsheet format, but use a relational database.

3.7. Keep a record of any freeze-thaw that occurs with a sample for any reason.

3.8. Track any change of location of a sample, including sending a sample out to an assay lab for processing.

3.9. Track any new samples created from the original sample (i.e., a sub-sample) in the same manner as described above. Ensure that each sub-sample/aliquot is labelled as described in section 2, with a unique ID.

**4. Freezer check**

4.1.1. ***Standard collection:*** Check freezers bi-weekly and keep a written-log of checks. Have alarm systems setup on all freezers in addition to human bi-weekly checks.

4.1.2. ***Required minimum:*** Manually check freezers bi-weekly and keep a written log of checks.

**5. Data recording check list**

5.1. Record protocol, specifying which steps are adhered to (standard or minimum).

5.2. For each sample, record:

5.2.1. Date and time of collection (Date: __/__/__ and __:__am/pm).

5.2.2. Start time of sample processing in the laboratory (Date: __/__/__ and __:__am/pm).

5.2.3. Type and number of lesions prepared.

5.2.4. Date and time sample is stored into freezer (Date: __/__/__ and __:__am/pm).

5.2.5. Any variations or deviations from the SOP, problems, or issues.

5.2.6. Any freeze-thaw event that occurs with a sample for any reason.

5.7. Keep a log of bi-weekly freezer checks.

**Supplemental Appendix II:**

**Detailed standard operating procedure for the collection, processing and storage of eutopic endometrium samples**

**NOTES**

- This SOP does not cover safety procedures for the collection and processing of these samples and personnel must follow institutional biosafety guidelines**.**
- For guidance on surgical excision of eutopic endometrium biopsies, please refer to Becker *et al.*, (Becker *et al.*, 2014).
- For a summary version of this protocol with side-by-side standard vs. minimal protocol step comparisons, please see Supplemental Table 2.
- As this protocol applies to different processing and storage methods (e.g. the use of RNA stabilising solution; immediate snap freezing in theatre), keep a copy of the exact step-by-step protocol used in your lab.

***Processing and storage materials***

1. Biospecimen questionnaire (Supplemental Appendix V);
2. Log sheet to record sample-related data;

*Frozen tissues:*

1. Labels suitable for long-term freezer storage, and IDs printed using 2D barcoding.
2. Aliquot vials with screw top gasket closure; liquid nitrogen.
3. Buffers: phosphate buffered saline (PBS);
4. RNA stabilizer solution *(optional if planning RNA studies)*
5. Freezers: -80C or liquid nitrogen (LN_2_)

*Fixed tissues:*

1. Labels with IDs printed using 2D barcoding;
2. Neutral buffered formalin, universal molecular fixative, paraffin;
3. HistokinetteTM cassette

**1. Eutopic endometrium collection**

1.1 Pr_i_or to tissue collection at surgery, prepare as many of the materials required for storage of the samples and recording of the data (log sheet, storage vials and labels).

1.2. Preparation of vials/tubes for surgical collection *(NB. If samples are to be snap-frozen immediately in liquid nitrogen in/near theatre in their final storage vials, which is recommended, adopt the final labelling guidelines in section 2 of this protocol).*

1.2.1. ***Standard collection:*** Pre-label each collection vial with a 2D barcode in addition to a human readable unique identifier, participant ID, date of collection and type of sample. Record on the log sheet the date and time of sample collection (Date: __/__/__ and __:__am/pm).

1.2.2. ***Required minimum:*** Pre-label each collection vial with a unique identifier, participant ID, date of collection, and type of sample. Record on the log sheet the date and time of sample collection (Date: __/__/__ and __:__am

1.3.1. ***Standard collection:*** Record whether eutopic endometrium is collected: wet weight and method used (curettage, endometrial sampling device, hysterectomy and hysteroscopy specimen and Tao brush^TM,^ (Cook, Bloomington, USA)).

1.3.2. ***Required minimum:*** Record whether eutopic endometrium is collected and method used (curettage, endometrial sampling device, and Tao brush ^TM^(Cook, Bloomington, USA)).

1.4.1. ***Standard collection:*** Collect eutopic endometrium before anaesthesia induction.

1.4.2. ***Required minimum****:* Record whether eutopic endometrium is collected 1) prior to anaesthesia, 2) after sedation but before anaesthesia, or 3) after anaesthesia.

1.5. Deposition of collected tissue samples.

1.5.1. ***Standard collection:*** Insert tissues in prepared, labelled vials/tubes and snap-freeze immediately in liquid nitrogen in/near theatre in their final storage vials.

1.5.2. ***Required minimum:*** Insert tissues in prepared, labelled vials/tubes and snap-freeze as soon as possible in liquid nitrogen in/near theatre in their final storage vials. If delays >15 minutes are expected, immerse in an RNA stabiliser solution if required.

**2. Sample processing**

2.1. Preparation of vials/tubes for collection and storage. Use labels and ink suitable for long-term freezer storage. Do not use laser printers or most ink-jet printers as the ink can crack and fall off the label when frozen at ultra-low temperature.

2.1.1. ***Standard collection****:* Pre-label the aliquot vials with the participant ID number followed by a unique aliquot ID number. Include date of sample creation and sample type. For example: ENDO-123456-U654321-EB: Center identifier (ENDO), participant ID (123456), unique aliquot vial ID (U654321), and sample type (EB for eutopic endometrium). Further, include the above information in human readable format and in a 2D barcode on the label.

2.1.2. ***Required minimum:*** Pre-label the aliquot vials with the participant ID followed by the sample aliquot number. Also include date of sample creation and sample type on the label. For example: ENDO-123456-EB-01: Center identifier (ENDO), participant ID (123456), type of sample (EB for eutopic endometrium), aliquot number (01).

2.2. Record time of starting sample processing on log sheet.

2.3. Rinse tissue with PBS before storage (except for RNA studies).

2.4. Weigh tissue samples. If tissue size and weight allows storage in separate pieces, prioritize in the following order before freezing for long-term storage.: 1^st^ snap freezing, 2^nd^ RNA stabilizing solution followed by freezing, 3^rd^ universal molecular fixative or formalin fixation. *NB. If RNA is of key interest, and time to freezing is likely to take more than 5 minutes, consider immediate immersion in an RNA stabilizer solution as the first priority.*

2.5. Fresh Tissues

2.5.1. ***Standard collection****:* Fresh tissue can be stored in fluid (eg. media or PBS) up to 24 hours at 4°C. Transfer to waterbath (37°C) to keep cells alive for cell culture.

2.5.2. ***Required minimum*:** Fresh tissue can be stored in fluid (eg. media or PBS) up to 24 hours at 4°C.

2.6. Frozen Tissues

2.6.1. ***Standard collection****:* Tissue samples should be stored in an aliquot vial with screw top gasket closure. Snap freeze in liquid nitrogen within 15 minutes, record time, and transfer to freezers for long-term storage (see section 3).

2.6.2. ***Required minimum****:* Tissue samples should be stored in an aliquot vial with screw top gasket closure. Snap freeze in liquid nitrogen as soon as possible, record time, and transfer to freezers for long-term storage (see section 3).

2.7. RNA stabilizing solution

[commercially available products: Allprotect Tissue Reagent® (Qiagen, Venlo, Netherlands); DNA / RNA Shield™ (Zymoresearch, Irvine, USA); ProtectRNA™ (Sigma-Aldrich, St.Louis, USA); RiboLock™ (Thermoscientific, Waltham, USA); RNAlater® (Qiagen, Venlo, Netherlands); Ambion® [RNAsecure™ Reagent](http://products.invitrogen.com/ivgn/product/AM7006) [(Life-technologies, Carlsbad, USA); SUPERase•In™ (Life-technologies, Carlsbad, USA); PAXgene Tissue Containers (Qiagen, Venlo, Netherlands)]](http://products.invitrogen.com/ivgn/product/AM2694)

Before immersion in RNA stabilizing solution, cut large tissue samples to size according to manufacturer’s protocol.

2.7.1. ***Standard collection****:* Tissue samples should be stored in an aliquot vial with screw top gasket closure containing RNA stabilizing solution within 15 minutes of collection, and stored at 4°C for 24 hours prior to freezing (see section 3).

2.7.2. ***Required minimum****:* Tissue samples should be stored in an aliquot vial with screw top gasket closure containing RNA stabilizing solution, and stored at room temperature for 24 hours prior to freezing (see section 3).

2.8. **Fixed tissues**

2.8.1. ***Standard collection****:* Tissue samples should be mounted flat in a Histokinette^TM^ cassette into 20ml of 10% neutral buffered formalin (NBF), within 15 minutes (record time). The sample is transported at room temperature or can be placed on ice. The tissue should remain in NBF for no more than 24 hours before transfer to graded alcohol solutions and paraffin embedding. Alternative fixatives include universal molecular fixative, which may better preserve RNA.

2.8.2. ***Required minimum****:* Tissue samples should be mounted flat in a Histokinette^TM^ cassette containing 20 ml of 10% neutral buffered formalin (NBF), within 1 hour (record time). The sample is transported at room temperature or can be placed on ice. The tissue should remain in NBF for no more than 24 hours before transfer to graded alcohol solutions and paraffin embedding. Alternative fixatives include universal molecular fixative, which may better preserve RNA.

**3. Storage and data recording**

3.1. Date and time of storage should be recorded on the log sheet. Also record the type, number of aliquots prepared.

3.1.1. ***Standard collection****:* Store samples in liquid nitrogen (LN_2_) freezers, as they have less temperature fluctuations than -80°C freezers.

3.1.2. ***Required minimum:*** Store samples in -80°C or lower freezers.

3.5. Record on the log sheet any variations or deviations from the SOP, problems, or issues (e.g. vial cracked during processing).

3.6. Record the location of each sample into the freezer including freezer number, rack, box, and spot in the box along with all other sample attributes in a database. If possible, avoid using a spreadsheet format, but use a relational database.

3.7. Keep a record of any freeze-thaw that occurs with a sample for any reason.

3.8. Track any change of location of a sample, including sending a sample out to an assay lab for processing.

3.9. Track any new samples created from the original sample (i.e., a sub-sample) in the same manner as described above. Ensure that each sub-sample/aliquot is labelled as described in section 2, with a unique ID.

**4. Freezer check**

4.1.1. ***Standard collection:*** Check freezers bi-weekly and keep a written-log of checks. Have alarm systems setup on all freezers in addition to human bi-weekly checks.

4.1.2. ***Required minimum:*** Manually check freezers bi-weekly and keep a written log of checks.

**5. Data recording check list**

5.1. Record protocol, specifying which steps are adhered to (standard or minimum).

5.2. For each sample, record:

5.2.1. Date and time of collection (Date: __/__/__ and __:__am/pm).

5.2.2. Start time of sample processing in the laboratory (Date: __/__/__ and __:__am/pm).

5.2.3. Record type and number of biopsies prepared.

5.2.4. Date and time sample is stored into freezer (Date: __/__/__ and __:__am/pm).

5.2.5. Any variations or deviations from the SOP, problems, or issues.

5.2.6. Any freeze-thaw event that occurs with a sample for any reason.

5.7. Keep a log of bi-weekly freezer checks.

**Supplemental Appendix III:**

**Detailed standard operating procedure for the collection, processing and storage of myometrium samples**

**NOTES**

- This SOP does not cover safety procedures for the collection and processing of these samples and personnel must follow institutional biosafety guidelines**.**
- For guidance on surgical excision of myometrium biopsies, please refer to Becker *et al.*, (Becker *et al.*, 2014). For a summary version of this protocol with side-by-side standard vs. minimal protocol step comparisons, please see Supplemental Table 3.
- As this protocol applies to different processing and storage methods (e.g. the use of RNA stabilising solution; immediate snap freezing in theatre), keep a copy of the exact step-by-step protocol used in your lab.

***Processing and storage materials***

1. Biospecimen questionnaire (Supplemental Appendix V);
2. Log sheet to record sample-related data;

*Frozen tissues:*

1. Labels suitable for long-term freezer storage, and IDs printed using 2D barcoding.
2. Aliquot vials with screw top gasket closure; liquid nitrogen.
3. Buffers: phosphate buffered saline (PBS);
4. RNA stabilizer solution *(optional if planning RNA studies)*
5. Freezers: -80C or liquid nitrogen (LN_2_)

*Fixed tissues:*

1. Labels with IDs printed using 2D barcoding;
2. Neutral buffered formalin, universal molecular fixative, paraffin;
3. HistokinetteTM cassette
4. **Myometrium collection**

1.1 Pr_i_or to tissue collection at surgery, prepare as many of the materials required for storage of the samples and recording of the data (log sheet, storage vials and labels).

1.2. Preparation of vials/tubes for surgical collection *(NB. If samples are to be snap-frozen immediately in liquid nitrogen in/near theatre in their final storage vials, which is recommended, adopt the final labelling guidelines in section 2 of this protocol).*

1.2.1. ***Standard collection:*** Pre-label each collection vial with a 2D barcode in addition to a human readable unique identifier, participant ID, date of collection and type of sample. Record on the log sheet the date and time of sample collection (Date: __/__/__ and __:__am/pm).

1.2.2. ***Required minimum:*** Pre-label each collection vial with a unique identifier, participant ID, date of collection, and type of sample. Record on the log sheet the date and time of sample collection (Date: __/__/__ and __:__am/pm).

1.3. Deposition of collected tissue samples.

1.3.1. ***Standard collection:*** Insert tissues in prepared, labelled vials/tubes and snap-freeze immediately in liquid nitrogen in/near theatre in their final storage vials.

1.3.2. ***Required minimum:*** Insert tissues in prepared, labelled vials/tubes and snap-freeze as soon as possible in liquid nitrogen in/near theatre in their final storage vials. If delays >15 minutes are expected, immerse in an RNA stabiliser solution if required.

**2. Sample processing**

2.1. Preparation of vials/tubes for collection and storage. Use labels and ink suitable for long-term freezer storage. Do not use laser printers or most ink-jet printers as the ink can crack and fall off the label when frozen at ultra-low temperature.

2.1.1. ***Standard collection****:* Pre-label the aliquot vials with the participant ID number followed by a unique aliquot ID number. Include date of sample creation and sample type. For example: ENDO-123456-U654321-MB: Center identifier (ENDO), participant ID (123456), unique aliquot vial ID (U654321), and sample type (MB for myometium). Further, include the above information in human readable format and in a 2D barcode on the label.

2.1.2. ***Required minimum:*** Pre-label the aliquot vials with the participant ID followed by the sample aliquot number. Also include date of sample creation and sample type on the label. For example: ENDO-123456-MB-01: Center identifier (ENDO), participant ID (123456), type of sample (MB for myometrium), aliquot number (01).

2.2. Record time of starting sample processing on log sheet.

2.3. Rinse tissue with PBS (except for RNA studies).

2.4. Weigh tissue samples. If tissue size and weight allows storage in separate pieces, prioritize in the following order before freezing for long-term storage.: 1^st^ snap freezing, 2^nd^ RNA stabilizing solution followed by freezing, 3^rd^ universal molecular fixative or formalin fixation. *NB. If RNA is of key interest, and time to freezing is likely to take more than 5 minutes, consider immediate immersion in an RNA stabilizer solution as the first priority.*

2.5. Fresh Tissues

2.5.1. ***Standard collection****:* Fresh tissue can be stored in fluid (eg. media or PBS) up to 24 hours at 4°C. Transfer to waterbath (37°C) to keep cells alive for cell culture.

2.5.2. ***Required minimum*:** Fresh tissue can be stored in fluid (eg. media or PBS) up to 24 hours at 4°C.

2.6. Frozen Tissues

2.6.1. ***Standard collection****:* Tissue samples should be stored in an aliquot vial with screw top gasket closure. Snap freeze in liquid nitrogen within 15 minutes, record time, and transfer to freezers for long-term storage (see section 3).

2.6.2. ***Required minimum****:* Tissue samples should be stored in an aliquot vial with screw top gasket closure. Snap freeze in liquid nitrogen as soon as possible, record time, and transfer to freezers for long-term storage (see section 3).

2.7. RNA stabilizing solution

[commercially available products: Allprotect Tissue Reagent® (Qiagen, Venlo, Netherlands); DNA / RNA Shield™ (Zymoresearch, Irvine, USA); ProtectRNA™ (Sigma-Aldrich, St.Louis, USA); RiboLock™ (Thermoscientific, Waltham, USA); RNAlater® (Qiagen, Venlo, Netherlands); Ambion® [RNAsecure™ Reagent](http://products.invitrogen.com/ivgn/product/AM7006) [(Life-technologies, Carlsbad, USA); SUPERase•In™ (Life-technologies, Carlsbad, USA); PAXgene Tissue Containers (Qiagen, Venlo, Netherlands)]](http://products.invitrogen.com/ivgn/product/AM2694)

Before immersion in RNA stabilizing solution, cut large tissue samples to size according to manufacturer’s protocol.

2.7.1. ***Standard collection****:* Tissue samples should be stored in an aliquot vial with screw top gasket closure containing RNA stabilizing solution within 15 minutes of collection, and stored at 4°C for 24 hours prior to freezing (see section 3).

2.7.2. ***Required minimum****:* Tissue samples should be stored in an aliquot vial with screw top gasket closure containing RNA stabilizing solution, and stored at room temperature for 24 hours prior to freezing (see section 3).

2.8. Fixed Tissues

2.8.1. ***Standard collection****:* Tissue samples should be mounted flat in a Histokinette^TM^ cassette into 20ml of 10% neutral buffered formalin (NBF), within 15 minutes (record time). The sample is transported at room temperature or can be placed on ice. The tissue should remain in NBF for no more than 24 hours before transfer to graded alcohol solutions and paraffin embedding. Alternative fixatives include universal molecular fixative, which may better preserve RNA.

2.8.2. ***Required minimum****:* Tissue samples should be mounted flat in a Histokinette^TM^ cassette containing 20 ml of 10% neutral buffered formalin (NBF), within 1 hour (record time). The sample is transported at room temperature or can be placed on ice. The tissue should remain in NBF for no more than 24 hours before transfer to graded alcohol solutions and paraffin embedding. Alternative fixatives include universal molecular fixative, which may better preserve RNA.

**3. Storage and data recording**

3.1. Date and time of storage should be recorded on the log sheet. Also record the type, number of aliquots prepared.

3.1.1. ***Standard collection****:* Store samples in liquid nitrogen (LN_2_) freezers, as they have less temperature fluctuations than -80°C freezers.

3.1.2. ***Required minimum:*** Store samples in -80°C or lower freezers.

3.5. Record on the log sheet any variations or deviations from the SOP, problems, or issues (e.g. vial cracked during processing).

3.6. Record the location of each sample into the freezer including freezer number, rack, box, and spot in the box along with all other sample attributes in a database. If possible, avoid using a spreadsheet format, but use a relational database.

3.7. Keep a record of any freeze-thaw that occurs with a sample for any reason.

3.8. Track any change of location of a sample, including sending a sample out to an assay lab for processing.

3.9. Track any new samples created from the original sample (i.e., a sub-sample) in the same manner as described above. Ensure that each sub-sample/aliquot is labelled as described in section 2, with a unique ID.

**4. Freezer check**

4.1.1. ***Standard collection:*** Check freezers bi-weekly and keep a written-log of checks. Have alarm systems setup on all freezers in addition to human bi-weekly checks.

4.1.2. ***Required minimum:*** Manually check freezers bi-weekly and keep a written log of checks.

**5. Data recording check list**

5.1. Record protocol, specifying which steps are adhered to (standard or minimum).

5.2. For each sample, record:

5.2.1. Date and time of collection (Date: __/__/__ and __:__am/pm).

5.2.2. Start time of sample processing in the laboratory (Date: __/__/__ and __:__am/pm).

5.2.3. Record type and number of biopsies prepared.

5.2.4. Date and time sample is stored into freezer (Date: __/__/__ and __:__am/pm).

5.2.5. Any variations or deviations from the SOP, problems, or issues.

5.2.6. Any freeze-thaw event that occurs with a sample for any reason.

5.7. Keep a log of bi-weekly freezer checks.

**Supplemental Appendix IV:**

**Detailed standard operating procedure for the collection, processing and storage of peritoneum samples**

**NOTES**

- This SOP does not cover safety procedures for the collection and processing of these samples and personnel must follow institutional biosafety guidelines**.**
- For guidance on surgical excision of peritoneal biopsies, please refer to Becker *et al.*, (Becker *et al.*, 2014). For a summary version of this protocol with side-by-side standard vs. minimal protocol step comparisons, please see Supplemental Table 4.
- As this protocol applies to different processing and storage methods (e.g. the use of RNA stabilising solution; immediate snap freezing in theatre), keep a copy of the exact step-by-step protocol used in your lab.

***Processing and storage materials***

1. Biospecimen questionnaire (Supplemental Appendix V);
2. Log sheet to record sample-related data;

*Frozen tissues:*

1. Labels suitable for long-term freezer storage, and IDs printed using 2D barcoding.
2. Aliquot vials with screw top gasket closure; liquid nitrogen.
3. Buffers: phosphate buffered saline (PBS);
4. RNA stabilizer solution *(optional if planning RNA studies)*
5. Freezers: -80C or liquid nitrogen (LN_2_)

*Fixed tissues:*

1. Labels with IDs printed using 2D barcoding;
2. Neutral buffered formalin, universal molecular fixative, paraffin;
3. HistokinetteTM cassette

**1. Peritoneum collection**

1.1 Pr_i_or to tissue collection at surgery, prepare as many of the materials required for storage of the samples and recording of the data (log sheet, storage vials and labels).

1.2. Preparation of vials/tubes for surgical collection *(NB. If samples are to be snap-frozen immediately in liquid nitrogen in/near theatre in their final storage vials, which is recommended, adopt the final labelling guidelines in section 2 of this protocol).*

1.2.1. ***Standard collection:*** Pre-label each collection vial with a 2D barcode in addition to a human readable unique identifier, participant ID, date of collection and type of sample. Record on the log sheet the date and time of sample collection (Date: __/__/__ and __:__am/pm).

1.2.2. ***Required minimum:*** Pre-label each collection vial with a unique identifier, participant ID, date of collection, and type of sample. Record on the log sheet the date and time of sample collection (Date: __/__/__ and __:__am/pm).

1.3.1. ***Standard collection:*** Record whether peritoneum is collected: wet weight, method used (excised through sharp dissection, or Tao brush^TM,1^(Cook, Bloomington, USA)) and location [lateral on pelvic brim, other location in the pelvis (to be specified)].

1.3.2. ***Required minimum:*** Record whether peritoneum is collected: method used (excised by sharp dissection, laser, diathermy or cells collected using a Tao brush ^TM^(Cook, Bloomington, USA)) and location [lateral on pelvic brim, within Pouch of Douglas].

1.4. Deposition of collected tissue samples.

1.4.1. ***Standard collection:*** Insert tissues in prepared, labelled vials/tubes and snap-freeze immediately in liquid nitrogen in/near theatre in their final storage vials.

1.4.2. ***Required minimum:*** Insert tissues in prepared, labelled vials/tubes and snap-freeze as soon as possible in liquid nitrogen in/near theatre in their final storage vials. If delays >15 minutes are expected, immerse in an RNA stabiliser solution if required.

**2. Sample processing**

2.1. Preparation of vials/tubes for collection and storage. Use labels and ink suitable for long-term freezer storage. Do not use laser printers or most ink-jet printers as the ink can crack and fall off the label when frozen at ultra-low temperature.

2.1.1. ***Standard collection****:* Pre-label the aliquot vials with the participant ID number followed by a unique aliquot ID number. Include date of sample creation and sample type. For example: ENDO-123456-U654321-PB: Center identifier (ENDO), participant ID (123456), unique aliquot vial ID (U654321), and sample type (PB for Peritoneum). Further, include the above information in human readable format and in a 2D barcode on the label.

2.1.2. ***Required minimum:*** Pre-label the aliquot vials with the participant ID followed by the sample aliquot number. Also include date of sample creation and sample type on the label. For example: ENDO-123456-PB-01: Center identifier (ENDO), participant ID (123456), type of sample (PB for peritoneum), aliquot number (01).

2.2. Record time of starting sample processing on log sheet.

2.3. Rinse tissue with PBS (except for RNA studies).

2.4. Weigh tissue samples. If tissue size and weight allows storage in separate pieces, prioritize in the following order before freezing for long-term storage.: 1^st^ snap freezing, 2^nd^ RNA stabilizing solution followed by freezing, 3^rd^ universal molecular fixative or formalin fixation. *NB. If RNA is of key interest, and time to freezing is likely to take more than 5 minutes, consider immediate immersion in an RNA stabilizer solution as the first priority.*

2.5. Fresh Tissues

2.5.1. ***Standard collection****:* Fresh tissue can be stored in fluid (eg. media or PBS) up to 24 hours at 4°C. Transfer to waterbath (37°C) to keep cells alive for cell culture.

2.5.2. ***Required minimum*:** Fresh tissue can be stored in fluid (eg. media or PBS) up to 24 hours at 4°C.

2.6. Frozen Tissues

2.6.1. ***Standard collection****:* Tissue samples should be stored in an aliquot vial with screw top gasket closure. Snap freeze in liquid nitrogen within 15 minutes, record time, and transfer to freezers for long-term storage (see section 3).

2.6.2. ***Required minimum****:* Tissue samples should be stored in an aliquot vial with screw top gasket closure. Snap freeze in liquid nitrogen as soon as possible, record time, and transfer to freezers for long-term storage (see section 3).

2.7. RNA stabilizing solution

[commercially available products: Allprotect Tissue Reagent® (Qiagen, Venlo, Netherlands); DNA / RNA Shield™ (Zymoresearch, Irvine, USA); ProtectRNA™ (Sigma-Aldrich, St.Louis, USA); RiboLock™ (Thermoscientific, Waltham, USA); RNAlater® (Qiagen, Venlo, Netherlands); Ambion® [RNAsecure™ Reagent](http://products.invitrogen.com/ivgn/product/AM7006) [(Life-technologies, Carlsbad, USA); SUPERase•In™ (Life-technologies, Carlsbad, USA); PAXgene Tissue Containers (Qiagen, Venlo, Netherlands)]](http://products.invitrogen.com/ivgn/product/AM2694)

Before immersion in RNA stabilizing solution, cut large tissue samples to size according to manufacturer’s protocol.

2.7.1. ***Standard collection****:* Tissue samples should be stored in an aliquot vial with screw top gasket closure containing RNA stabilizing solution within 15 minutes of collection, and stored at 4°C for 24 hours prior to freezing (see section 3).

2.7.2. ***Required minimum****:* Tissue samples should be stored in an aliquot vial with screw top gasket closure containing RNA stabilizing solution, and stored at room temperature for 24 hours prior to freezing (see section 3).

2.8. Fixed Tissues

2.8.1. ***Standard collection****:* Tissue samples should be mounted flat in a Histokinette^TM^ cassette into 20ml of 10% neutral buffered formalin (NBF), within 15 minutes (record time). The sample is transported at room temperature or can be placed on ice. The tissue should remain in NBF for no more than 24 hours before transfer to graded alcohol solutions and paraffin embedding. Alternative fixatives include universal molecular fixative, which may better preserve RNA.

2.8.2. ***Required minimum****:* Tissue samples should be mounted flat in a Histokinette^TM^ cassette containing 20 ml of 10% neutral buffered formalin (NBF), within 1 hour (record time). The sample is transported at room temperature or can be placed on ice. The tissue should remain in NBF for no more than 24 hours before transfer to graded alcohol solutions and paraffin embedding. Alternative fixatives include universal molecular fixative, which may better preserve RNA.

**3. Storage and data recording**

3.1. Date and time of storage should be recorded on the log sheet. Also record the type, number of aliquots prepared.

3.1.1. ***Standard collection****:* Store samples in liquid nitrogen (LN_2_) freezers, as they have less temperature fluctuations than -80°C freezers.

3.1.2. ***Required minimum:*** Store samples in -80°C or lower freezers.

3.5. Record on the log sheet any variations or deviations from the SOP, problems, or issues (e.g. vial cracked during processing).

3.6. Record the location of each sample into the freezer including freezer number, rack, box, and spot in the box along with all other sample attributes in a database. If possible, avoid using a spreadsheet format, but use a relational database.

3.7. Keep a record of any freeze-thaw that occurs with a sample for any reason.

3.8. Track any change of location of a sample, including sending a sample out to an assay lab for processing.

3.9. Track any new samples created from the original sample (i.e., a sub-sample) in the same manner as described above. Ensure that each sub-sample/aliquot is labelled as described in section 2, with a unique ID.

**4. Freezer check**

4.1.1. ***Standard collection:*** Check freezers bi-weekly and keep a written-log of checks. Have alarm systems setup on all freezers in addition to human bi-weekly checks.

4.1.2. ***Required minimum:*** Manually check freezers bi-weekly and keep a written log of checks.

**5. Data recording check list**

5.1. Record protocol, specifying which steps are adhered to (standard or minimum).

5.2. For each sample, record:

5.2.1. Date and time of collection (Date: __/__/__ and __:__am/pm).

5.2.2. Start time of sample processing in the laboratory (Date: __/__/__ and __:__am/pm).

5.2.3. Record type of biopsy prepared.

5.2.4. Date and time sample is stored into freezer (Date: __/__/__ and __:__am/pm).

5.2.5. Any variations or deviations from the SOP, problems, or issues.

5.2.6. Any freeze-thaw event that occurs with a sample for any reason.

5.7. Keep a log of bi-weekly freezer checks.

Footnote:

^1^For the Tao brush^TM^(Cook, Bloomington, USA), a sweep is made over the peritoneal surface, rotating the brush to obtain surface epithelial cells carefully avoiding the ovary, and subsequently immersing in warm (close to 37°C) HOSE medium [40% Medium 199, 40% MCDB 105 supplemented with 15% fetal bovine serum, 0.5% penicillin-streptomycin mix and 1% L-glutamine] while agitating to remove the cells.

**Supplemental Appendix V:**

**EPHect Biospecimen Collection Form** (to be completed by research nurse)

Date and time sample collected: (DD/MM/YYYY) ___ / ___ / _______ Time: ____:____ AM/PM

What was the first day of your last menstrual period? (DD/MM/YYYY) ___ / ___ / _______

Are your periods regular? (Predictable within one week)

🞏 Yes 🞏 No

Specify range of days : [regular range: 21-35days]

If you have not had a menstrual period in the past 90 days, please tell us why:

##### 🞎 Taking hormones continuously (*e.g. the Pill, injections, Mirena, HRT*)

##### 🞎 Pregnant

##### 🞎 Breastfeeding

##### 🞎 Unsure

##### 🞎 Other (*Please describe*) ________________________________________________________

Are you currently having a menstrual period/vaginal bleeding *(including spotting for which you only need a panty liner)*?

🞎 No

🞎 Yes, menstrual period

🞎 Yes, irregular bleeding/spotting

Do you currently have a coil [IUD] in place?

🞎 No

🞎 Yes → If yes, what kind of IUD? 🞎 Progesterone containing IUD (Mirena)

🞎 Other coil/intrauterine device

When was the last time you had something to eat?

____ : ____ am/pm 🞎 Today 🞎 Yesterday

When was the last time you had something to drink (other than plain water) and what did you drink?

____ : ____ am/pm 🞎 Today 🞎 Yesterday

____________________________________________________________________________

**Clinical Measurements:**

Height: ______ in cm, or ______ in inches

Weight: ______ in kg, or ______ in pounds

Hip circumference: ______ in cm, or ______ in inches

Waist circumference: ______ in cm, or ______ in inches

*See NHANES III video guidelines on how to take measurements (also included as text in the Supplementary Appendix VIII in Rahmioglu et al., (20)):* https://www.youtube.com/watch?v=KacU_TW50Zo

**If saliva samples are being collected:**

Please indicate whether or not you have used the following in the last 24 hours and what time you used each item.

| Toothpaste | 🞎 No 🞎 Yes → | ___ : ___ AM/PM 🞎 Today 🞎 Yesterday |
| --- | --- | --- |
| Gum | 🞎 No 🞎 Yes → | ___ : ___ AM/PM 🞎 Today 🞎 Yesterday |
| Cigarettes | 🞎 No 🞎 Yes → | ___ : ___ AM/PM 🞎 Today 🞎 Yesterday |
| Alcohol | 🞎 No 🞎 Yes → | ___ : ___ AM/PM 🞎 Today 🞎 Yesterday |

In the past 24 hours have you eaten:

Spicy food? 🞎 No 🞎 Yes

Fish? 🞎 No 🞎 Yes

**If urine samples are being collected:**

When did you last urinate (prior to providing the sample)?

____ : ____ am/pm 🞎 Today 🞎 Yesterday

What time was the urine sample produced?

____ : ____ am/pm

Is this urine sample your first morning void?

🞎 No

🞎 Yes → If yes, did you get up during the night to urinate? 🞎 No 🞎 Yes

In collecting this sample, did you follow a clean catch protocol?

🞎 No

🞎 Yes

**If undergoing an operation:**

Was any pre-med taken before blood, urine, saliva, endometrial fluid and eutopic endometrium/ myometrium collection? (NB. EPHect recommends sample taking prior to pre-med administration)

🞏 No

🞏 Yes

If yes, tick which samples were taken after pre-med administration:

🞏Blood 🞏Urine 🞏Saliva 🞏Endometrial fluid

🞏Eutopic endometrium/ myometrium

Time pre-med was administered: ____am/pm

Please specify the type of pre-med was administered _____________________________

Was anaesthetic administered before blood, endometrial fluid and eutopic endometrium collection?

🞏 No

🞏 Yes

If yes, tick which samples were taken after anaesthesia administration:

🞏 Blood 🞏 Endometrial fluid 🞏 Eutopic endometrium

If yes, time anaesthetic was administered: ____am/pm

Please specify the type of pre-med was administered: __________________________

**Method(s) of excision:**

Ectopic endometrium

🞏 Electrosurgery

🞏 Harmonic scalpel

🞏 Laser [CO_2_, NdYag and others]

🞏 Cold scissors/scapels

Eutopic endometrium

🞏 Endometrial sampling device

🞏 Curettage with cervical dilation

🞏 Brushing

Myometrium

🞏 Laser [CO_2_, NdYag and others]

🞏 Electrosurgery

🞏 Cold scissors/scapels

🞏 TruCut biopsy

Peritoneum

🞏 laser [CO_2_, NdYag and others]

🞏 Electrosurgery

🞏 Ultrasound energy

🞏 Harmonic scalpel

🞏 Cold scissors/scapels

🞏 Brushing

**Method(s) of collection:**

Peritoneal fluid

🞏 No lavage. Amount collected ___ml

🞏 Lavage method with 10ml sterile saline solution. Amount of peritoneal lavage fluid (PLF) ___ml

Endometrial fluid

🞏 No lavage. Amount collected ___ml

🞏 Lavage method with 4ml sterile saline solution. Amount of uterine lavage fluid (ULF) ___ml

**Use of prescription drugs, non-prescription drugs, vitamins or supplements** **in the past 30 days.**

| **Type of drug** | **Have you ever taken this drug every day for over a month?** | **At what age did you first take this drug every day for over a month?** | **In total, how many years you have taken this drug? Please estimate, and enter “0 total years” if less than 1 year.** | **Are you currently taking this drug every day?** | **Please write down the specific name of the drug you have used most recently if known:** |
| --- | --- | --- | --- | --- | --- |
| **PRESCRIPTION DRUGS** | ✓ *if yes* | *Age 1^st^* | *Years taken:* | ✓ *if yes* | *Name of drug:* |
| a. Hormonal medications | 🞎 | __ __ | __ __ | 🞎 |  |
| Birth control pill | 🞎 | __ __ | __ __ | 🞎 |  |
| Progestin injection/shot | 🞎 | __ __ | __ __ | 🞎 |  |
| Transdermal patch/dot | 🞎 | __ __ | __ __ | 🞎 |  |
| Vaginal ring | 🞎 | __ __ | __ __ | 🞎 |  |
| Progesterone containing coil/IUD | 🞎 | __ __ | __ __ | 🞎 |  |
| Hormonal implant | 🞎 | __ __ | __ __ | 🞎 |  |
| Oral progestins to regulate cycle | 🞎 | __ __ | __ __ | 🞎 |  |
| GnRH agonist infection/shot | 🞎 | __ __ | __ __ | 🞎 |  |
| Norethindrone acetate | 🞎 | __ __ | __ __ | 🞎 |  |
| Danazol | 🞎 | __ __ | __ __ | 🞎 |  |
| Hormone replacement therapy (HRT) | 🞎 | __ __ | __ __ | 🞎 |  |
| Other: ……………………………………………… | 🞎 | __ __ | __ __ | 🞎 |  |
| b. Pain medications | 🞎 | __ __ | __ __ | 🞎 |  |
| Paracetamol/acetaminophen | 🞎 | __ __ | __ __ | 🞎 |  |
| Aspirin | 🞎 | __ __ | __ __ | 🞎 |  |
| Ibuprofen | 🞎 | __ __ | __ __ | 🞎 |  |
| COX-2 inhibitors (e.g. celebrex, vioxx) | 🞎 | __ __ | __ __ | 🞎 |  |
| Other anti-inflammatory analgesics  (e.g. naproxen, mefanamic acid, aleve,  naprosyn, relafen, keoprofen, anaprox) | 🞎 | __ __ | __ __ | 🞎 |  |
| Narcotic analgesics (e.g. hydrocodone+  paracetamol, codeine, morphine) | 🞎 | __ __ | __ __ | 🞎 |  |
| Muscle relaxants (e.g. diazepam/  temazepam, buscopan) | 🞎 | __ __ | __ __ | 🞎 |  |
| Other: ……………………………………………… | 🞎 | __ __ | __ __ | 🞎 |  |
| c. Diuretic (water pill) | 🞎 | __ __ | __ __ | 🞎 |  |
| d. Diabetic tablets | 🞎 | __ __ | __ __ | 🞎 |  |
| e. Insulin | 🞎 | __ __ | __ __ | 🞎 |  |
| f. Thyroid drugs | 🞎 | __ __ | __ __ | 🞎 |  |
| g. Drugs for epilepsy | 🞎 | __ __ | __ __ | 🞎 |  |
| h. Sleeping tablets / tranquilisers | 🞎 | __ __ | __ __ | 🞎 |  |
| i. Anti-depressants | 🞎 | __ __ | __ __ | 🞎 |  |
| j. Other drugs to treat mental illness | 🞎 | __ __ | __ __ | 🞎 |  |
| k. Drugs for osteoporosis (“brittle bones”) | 🞎 | __ __ | __ __ | 🞎 |  |
| l. Drugs for rheumatoid arthritis | 🞎 | __ __ | __ __ | 🞎 |  |
| m. Antibiotics for a month or more | 🞎 | __ __ | __ __ | 🞎 |  |
| n. Antacids | 🞎 | __ __ | __ __ | 🞎 |  |
| o. Drugs for stomach ulcer / gastritis | 🞎 | __ __ | __ __ | 🞎 |  |
| p. Drugs for high cholesterol | 🞎 | __ __ | __ __ | 🞎 |  |
| q. Drugs for allergies (antihistamines) | 🞎 | __ __ | __ __ | 🞎 |  |
| r. Steroids (oral, inhaled, or nasal) | 🞎 | __ __ | __ __ | 🞎 |  |
| s. Chemotherapy for cancer | 🞎 | __ __ | __ __ | 🞎 |  |
| t. Tamoxifen for cancer | 🞎 | __ __ | __ __ | 🞎 |  |
| u. Blood pressure drugs | 🞎 | __ __ | __ __ | 🞎 |  |
| v. Drugs for angina (chest pain) | 🞎 | __ __ | __ __ | 🞎 |  |
| w. Other drugs for a heart condition | 🞎 | __ __ | __ __ | 🞎 |  |
| x. Inhaler for asthma | 🞎 | __ __ | __ __ | 🞎 |  |
| y. Warfarin / heparin to thin blood | 🞎 | __ __ | __ __ | 🞎 |  |
| z. Migraine tablets/injections | 🞎 | __ __ | __ __ | 🞎 |  |
| Other 1: ……………………………. | 🞎 | __ __ | __ __ | 🞎 |  |
| Other 2: …………………………….. | 🞎 | __ __ | __ __ | 🞎 |  |
| Other 3: …………………………….. | 🞎 | __ __ | __ __ | 🞎 |  |
| Other 4: …………………………….. | 🞎 | __ __ | __ __ | 🞎 |  |
| Other 5: …………………………….. | 🞎 | __ __ | __ __ | 🞎 |  |

| **Type of drug** | **Have you taken this drug in the past 30 days?** | **Have you taken this drug in the past 48 hours**  **(2 days)?** | **In the past 30 days, on how many days have you taken this drug?** | **Please write down the specific name of the drug**  **if known:** |
| --- | --- | --- | --- | --- |
| **NON-PRESCRIPTION DRUGS** | *✓ if yes* | *✓ if yes* | *Number of days:* | *Name of drug:* |
| a. Aspirin | 🞎 | 🞎 | __ __ days |  |
| b. Paracetamol | 🞎 | 🞎 | __ __ days |  |
| c. Ibuprofen | 🞎 | 🞎 | __ __ days |  |
| d. Other anti-inflammatory analgesics (e.g. naproxen) | 🞎 | 🞎 | __ __ days |  |
| e. Herbal pain medication: ……………….. | 🞎 | 🞎 | __ __ days |  |
| f. Other pain medication: ………………….. |  |  |  |  |
| g. Migraine tablets | 🞎 | 🞎 | __ __ days |  |
| h. Antihistamine for allergies | 🞎 | 🞎 | __ __ days |  |
| i. Cold medicine / lemsip | 🞎 | 🞎 | __ __ days |  |
| j. Decongestant | 🞎 | 🞎 | __ __ days |  |
| k. Cough syrup | 🞎 | 🞎 | __ __ days |  |
| l. Antacids | 🞎 | 🞎 | __ __ days |  |
| m. Sleeping tablets | 🞎 | 🞎 | __ __ days |  |
| n. Eye drops | 🞎 | 🞎 | __ __ days |  |
| o. Vaginal thrush treatments (cream or tablets) | 🞎 | 🞎 | __ __ days |  |
| p. Cystitis treatments / cymalon | 🞎 | 🞎 | __ __ days |  |
| q. Mouth ulcer treatments | 🞎 | 🞎 | __ __ days |  |
| r. Nicotine replacement treatments | 🞎 | 🞎 | __ __ days |  |
| Other 1:......................................... | 🞎 | 🞎 | __ __ days |  |
| Other 2:......................................... | 🞎 | 🞎 | __ __ days |  |
| Other 3:......................................... | 🞎 | 🞎 | __ __ days |  |
| Other 4:......................................... | 🞎 | 🞎 | __ __ days |  |
| Other 5:......................................... | 🞎 | 🞎 | __ __ days |  |
|  |  |  |  |  |
| **VITAMINS & SUPPLEMENTS** |  |  |  |  |
| #1: ................................................. | 🞎 | 🞎 | __ __ days |  |
| #2: ................................................. | 🞎 | 🞎 | __ __ days |  |
| #3: ................................................. | 🞎 | 🞎 | __ __ days |  |
| #4: ................................................. | 🞎 | 🞎 | __ __ days |  |
| #5: ................................................. | 🞎 | 🞎 | __ __ days |  |
| #6: ................................................. | 🞎 | 🞎 | __ __ days |  |
| #7: ................................................. | 🞎 | 🞎 | __ __ days |  |
| #8: ................................................. | 🞎 | 🞎 | __ __ days |  |
| #9: ............................................... | 🞎 | 🞎 | __ __ days |  |
| #10: ............................................... | 🞎 | 🞎 | __ __ days |  |
